# Insulin promotes Rip11 accumulation at the plasma membrane by inhibiting a dynamin- and PI3-kinase-dependent, but Akt-independent, internalisation event

**DOI:** 10.1016/j.cellsig.2015.10.014

**Published:** 2016-01

**Authors:** Frédéric Boal, Lorna R. Hodgson, Sam E. Reed, Sophie E. Yarwood, Victoria J. Just, David J. Stephens, Mary W. McCaffrey, Jeremy M. Tavaré

**Affiliations:** a School of Biochemistry, University of Bristol, Bristol, BS8 1TD, UK; b Molecular Cell Biology Laboratory, School of Biochemistry and Cell Biology, Biosciences Institute, University College Cork, Cork, Ireland

**Keywords:** Insulin, Rip11, Rab, Recycling, Endocytosis

## Abstract

Rip11 is a Rab11 effector protein that has been shown to be important in controlling the trafficking of several intracellular cargoes, including the fatty acid transporter FAT/CD36, V-ATPase and the glucose transporter GLUT4. We have previously demonstrated that Rip11 translocates to the plasma membrane in response to insulin and here we examine the basis of this regulated phenomenon in more detail. We show that Rip11 rapidly recycles between the cell interior and surface, and that the ability of insulin to increase the appearance of Rip11 at the cell surface involves an inhibition of Rip11 internalisation from the plasma membrane. By contrast the hormone has no effect on the rate of Rip11 translocation towards the plasma membrane. The ability of insulin to inhibit Rip11 internalisation requires dynamin and class I PI3-kinases, but is independent of the activation of the protein kinase Akt; characteristics which are very similar to the mechanism by which insulin inhibits GLUT4 endocytosis.

## Highlights

• Rip11 translocates constitutively to the plasma membrane on Rab11-vesicles.
• Inhibition of Rip11 internalisation by insulin requires dynamin.
• Rip11 accumulation at the plasma membrane is PI3-K dependent but Akt-independent.

## 1 Introduction

The Rab family of small GTPases controls a multitude of trafficking pathways in eukaryotic cells [1], [2]. They cycle between an inactive GDP-bound state and an active GTP-bound state, in which they interact with different specialised protein partners that can dictate their subcellular localisation or even direct their function(s) [3]. Among the Rab protein family, several Rabs have been shown to regulate trafficking steps of the endosomal pathway. For instance, Rab5 and Rab7 are involved in the endocytic trafficking of cargoes and sorting through the early-to-late endosomes and lysosomes [4], [5], [6], Rab4 is involved in the direct recycling of the transferrin receptor (TfnR) from the early/sorting endosome [7], and Rab11 is involved in its indirect recycling from the juxtanuclear recycling compartment back to the plasma membrane [8].

Several Rab11-interacting proteins have been identified on the basis of the presence of a conserved Rab11-binding domain (RBD) [9], [10], [11], [12]. This family of proteins has been termed the Rab11 family of interacting proteins (Rab11–FIPs). Among this family, the class I Rab11–FIPs consist of Rab11–FIP1C (RCP, Rab coupling protein), Rab11–FIP2, and Rab11–FIP5 (Gaf1, pp75 or Rip11 as used in this study). They are predominantly localised to the endosomal recycling compartment, and are characterised by the presence of an N-terminal C2 domain and a C-terminal coiled-coil region encompassing the RBD. It has been shown that this coiled-coil region mediates dimerisation of the Rab11–FIPs [13], and their N-terminal C2 domain has been shown to bind to phosphoinositides (PtdIns) [14].

Among the class I Rab11–FIPs, the role of Rip11 in the recycling of transferrin has been well characterised. There is evidence that Rip11 functions in trafficking from early endosomes to juxtanuclear recycling endosomes (as suggested in [15]) and in the trafficking of cargo between the endosomal recycling compartment and the plasma membrane (as suggested in [14], [16]). The latter proposal is consistent with the reported role of Rip11 in the polarised transport of proteins from apical endosomes to the apical plasma membrane [17], the trafficking of the fatty acid transporter FAT/CD36 [18], the regulation of insulin granule exocytosis in pancreatic β-cells [19], the translocation of the V-ATPase from an intracellular pool to the plasma membrane in response to acidosis in salivary duct epithelial cells [20] and, as we have previously demonstrated, the insulin-stimulated trafficking of the glucose transporter GLUT4 to the plasma membrane of adipocytes [21].

Rip11 itself translocates from an intracellular compartment(s) to the plasma membrane in response to insulin and phorbol esters [14], [21]. In response to phorbol esters this translocation requires the presence of an intact Rip11 C2 domain, a region which binds phosphoinositides and phosphatidic acid in vitro [14]. Insulin-induced translocation is specific to Rip11, as it is not seen with any of the other class I FIPs [21]. However, the mechanism(s) by which these stimuli increase the translocation of Rip11 to the plasma membrane is not yet understood.

We previously reported that the effect of insulin on the translocation of a GFP–Rip11 chimera to the plasma membrane can be inhibited by wortmannin, a PI3-kinase inhibitor, and mimicked through over-expression of a constitutively-active mutant of Akt (Myr–Akt) [21]. This suggests that the phenomenon is PI3-kinase and Akt-dependent, however wortmannin is an inhibitor of all three classes of PI3-kinases [22], [23] including the Class II and III isoforms that generate phosphatidylinositol 3-phosphate on intracellular vesicles. Furthermore, since our original study was published a highly selective Akt inhibitor, MK2206 [24] has become available making it easier to explore the acute regulation of the phenomenon by Akt. The trafficking mechanism deployed by Rip11 in response to insulin is also not well understood. Given the previously identified role of Rip11 in GLUT4 translocation, together with our observation that Rip11 and GLUT4 colocalise on a small proportion of intracellular vesicles, we initially reasoned that Rip11 might translocate to the plasma membrane by ‘piggy-backing’ on GLUT4 vesicles. Thus in this study we examined both the signalling and trafficking mechanisms involved in more detail.

## 2 Materials and methods

### 2.1 Materials

Phosphatidylcholine (PC) and phosphatidylethanolamine (PE) were from Aventi Polar Lipids.

All other materials and reagents were from Sigma unless otherwise stated. Plasmids encoding mCherry–Rab11 and mCherry–Rab4 were kindly provided by Peter Cullen (University of Bristol, Bristol, UK).

### 2.2 Molecular cloning

The mutations in the Rip11 C2 domain were generated by gene synthesis (Genscript), based on the human sequence of Rip11 (amino acids 2–221; GenBank accession number AF334812). Codon optimization was undertaken essentially to decrease the %GC content and to facilitate subsequent cloning and site-directed mutagenesis. The wild-type and R52A/K54A mutant fragments were cloned in frame in the pGEX3X and peGFP-C1 vectors. To generate the full-length constructs (i.e. 2–653), the corresponding fragments 2–221 (WT or R52A/K54A) were excised from the peGFP-C1 using *Bam*HI and subcloned in frame into the C-terminal part of the Rip11 backbone (219–653) in the peGFP vector described in [14], generating a GFP–Rip11 2–653 construct. This construct was then excised from the peGFP-C1 vector and subcloned into the pmCherry-C1 vector to generate mCherry–Rip11.

### 2.3 Cell culture

3T3–L1 adipocytes were cultured, electroporated and treated as described before [21]. Stable cell lines were obtained by lentiviral-mediated transduction of undifferentiated 3T3–L1 by standards methods as described in [25], [26]. Lentiviral plasmids were gifts of Dr. Giles Cory (University of Exeter, Exeter, UK).

### 2.4 Live cell imaging and immunofluorescence

For immunofluorescence, cells grown on glass coverslips were treated as indicated in figure legends, and fixed with 4% paraformaldehyde for 20 min. Coverslips were mounted in Mowiol and imaged on a Leica TCS-SP5 AOBS scanning confocal microscope.

Total internal reflection fluorescence (TIRF) microscopy of live cells was performed at 37 °C on an AM TIRF multi-colour system (Leica) attached to a DMI 6000 inverted epifluorescence microscope (Leica) using a 100 × oil lens (NA = 1.46). The imaging medium consisted of phenol red-free DMEM/F12 (11,039, Invitrogen). Images were captured using an EM-CCD camera (Hamamatsu) between approx. 2–3 fps at a penetration depth of 90 nm. EGFP was excited at a wavelength of 488 nm and emission wavelengths were collected between 507 and 543 nm; mCherry was excited at a wavelength of 561 nm and emission wavelengths were collected between 584 and 616 nm. When required, cells were treated with 86 nM insulin on the microscope using a home-made perfusion system.

For inhibition of endocytosis, 3T3–L1 adipocytes stably expressing GFP–Rip11 WT were seeded on glass coverslips, and serum-starved for 3 h. The cells were then treated with vehicle (control), insulin (87 nM), Mitmab (10 or 40 μΜ) for 20 min at 37 °C. Alexa-Fluor™-633-transferrin (Invitrogen) was added for 20 min at 37 °C in the continuous presence of the drugs. The cells were then washed in PBS and PFA-fixed.

The amount of Rip11 at the cell surface was calculated using Volocity (PerkinElmer) by manually drawing around individual cells and calculating the fluorescence intensity from the whole cell, and then automatically contracting the region of interest by a set number of pixels in order to exclude the plasma membrane. The intensity within the inner ring was subtracted from the outer ring to give the amount of Rip11 within the plasma membrane and this value was normalised to total cellular levels of Rip11 (cell surface/total).

All images used in this manuscript were processed using Photoshop 6.0 (Adobe) and montages generated using Adobe Illustrator (Adobe). In Fig. 3 the images were subjected to contrast enhancement for illustrative purposes only, with all images being processed for display in an identical manner.

### 2.5 Recombinant protein purification

Recombinant GST-fusion proteins were produced and purified as described before [27]. Briefly, *Escherichia coli* BL21(DE3) (Agilent Technologies) were transformed by pGEX3X vectors containing the coding sequence for the Rip11 C2 domain (residues 2–221, WT or PI mutant) and protein expression was induced by adding 0.4 mM IPTG for 3 h at 30 °C. Bacteria were pelleted by centrifugation, washed with ice-cold phosphate buffered saline (PBS), and lysed by sonication in buffer [PBS, 0.4 mM EDTA, 2 mM DTT, 1 mM ATP, 1% Triton X100, 10 U/ml DNase I, Proteases Inhibitors Cocktail V (Calbiochem)]. The lysate was clarified by centrifugation at 10,000 g for 10 min at 4 °C, and incubated with gluthatione-Sepharose beads (GE Healthcare). The beads were washed extensively in buffer [PBS, 0.4 mM EDTA, 1% Triton X-100] and then in buffer A [50 mM Hepes pH 7.4, 100 mM NaCl, 1 mM CaCl_2_, 1 mM MgCl_2_]. Recombinant proteins were eluted in buffer A supplemented with 10 mM reduced glutathione.

### 2.6 Protein–lipid overlay assay

Protein–lipid overlay assay was essentially done as described elsewhere [14]. Briefly, PIP Strips™ (Invitrogen — Molecular Probes) were blocked in buffer A plus 0.1% Tween 20 containing 3% BSA, incubated overnight at 4 °C with 1.5 μg/ml GST fusion proteins in buffer A plus 0.1% Tween 20 plus 3% BSA and washed extensively. Bound GST recombinant proteins were detected using an HRP-coupled anti-GST antibody (B-14, Santa Cruz Biotechnology Inc).

### 2.7 Neutral phospholipid binding assay

Neutral phospholipid binding assay was essentially done according to [16]. Briefly, PC:PE liposomes in a ratio of 80:20 were made by sonication, incubated in Buffer A + 1 mM DTT for 30 min at RT with recombinant GST or GST–Rip11 C2 domain (WT or PI mutant). After sedimentation by ultracentrifugation at 70,000 rpm for 10 min at 4 °C in a TLA-100 Beckman rotor, the supernatants were collected and the pellets were solubilised in sample buffer, resolved by SDS-PAGE and the amount of recombinant proteins bound to the lipids was assessed by Western blotting, using an HRP-coupled anti-GST antibody.

### 2.8 Statistical analyses

Unless otherwise stated, all data are provided as a mean ± SEM from a minimum of three independent experiments. A two-tailed Student's t-test was used and a ‘p’ value of less than 0.05 taken as indicating a statistically relevant difference.

## 3 Results

### 3.1 Insulin stimulates Rip11 translocation to the plasma membrane in a PI3-kinase-dependent but Akt-independent manner

**Fig. 1:**
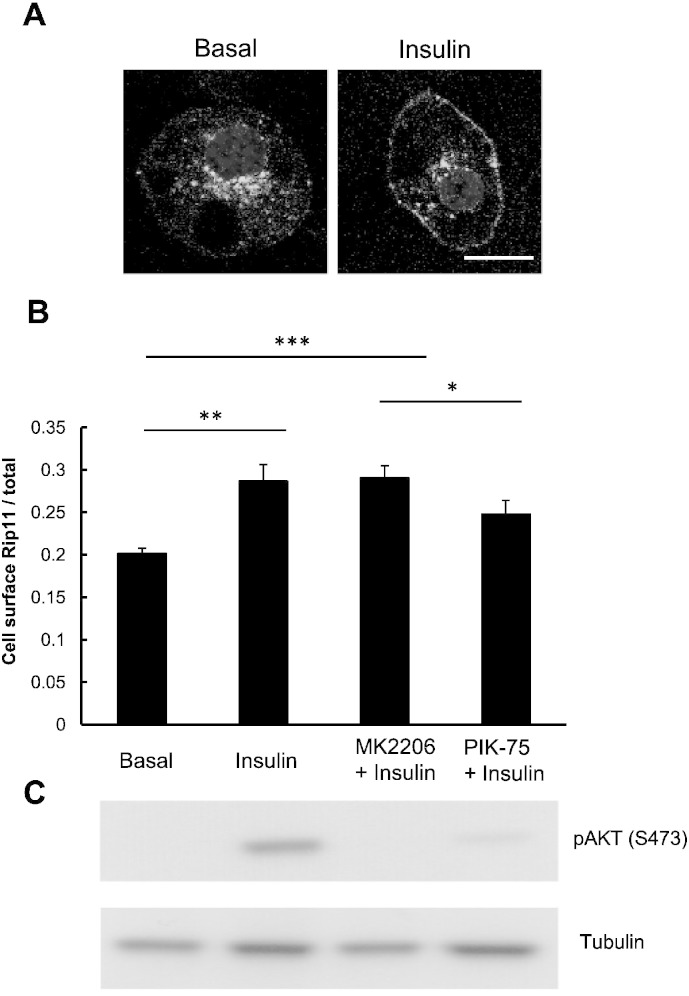
Rip11 translocates to the plasma membrane in a PI 3-kinase dependent but Akt-independent manner. 3T3–L1 adipocytes stably expressing GFP–Rip11 were serum starved and stimulated with or without insulin in the presence of the Akt inhibitor MK2206 or the PI 3-kinase inhibitor PIK-75, as indicated. (A) Representative confocal images of Rip11 localisation in the presence (right panel) and absence (left panel) of 87 nM insulin. Scale bar represents 17 μm. (B) Quantification of cell surface levels of Rip11 in the absence and presence of the PI 3-kinase inhibitor, PIK-75 (10 μM), or the Akt inhibitor, MK2206 (10 μM). Results are shown as mean ± SEM (N = 3, with at least 20 cells per condition (n), *p < 0.05, **p < 0.01,***p < 0.001 t-test). (C) Immunoblots are shown of 3T3–L1 lysates stably expressing GFP–Rip11 blotted with anti-pAKT (S473) and α-Tubulin (loading control) antibodies, having been treated under the same conditions as the cells in panel (B).

As exemplified in Fig. 1A (left panel), Rip11 is predominantly localised at intracellular membranes of 3T3–L1 adipocytes in the basal state but translocates to the plasma membrane in response to insulin. Quantification of the images shows that approx. 20% of Rip11 is found at the cell surface in basal cells and this rises to approx. 30% in the presence of insulin (Fig. 1B). The figure in the basal state is likely to be an over-estimate as the method we use in the calculation cannot easily discriminate between Rip11 in the plasma membrane itself, and that present in vesicles that immediately underlie the membrane (i.e. docked vesicles).

As shown in Fig. 1B, the insulin-stimulated translocation of GFP-tagged Rip11 to the plasma membrane was blocked by PIK-75, a small molecule p110α-selective PI 3-kinase inhibitor [28]. This occurred in parallel with an inhibition of Akt phosphorylation on serine 473 (Fig. 1C), an event well established to be regulated by Class I PI 3-kinase isoforms. By contrast, MK2206, a highly selective allosteric Akt inhibitor, had no effect on insulin-stimulated GFP–Rip11 translocation (Fig. 1B) despite almost completely inhibiting Akt Ser-473 phosphorylation (Fig. 1C). This data suggests that the ability of insulin to stimulate Rip11 translocation to the plasma membrane is dependent on the p110α class of PI 3-kinase but independent of the activation of the downstream kinase Akt.

### 3.2 Insulin does not affect the rate of fusion of Rip11 vesicles with the plasma membrane

To explore the trafficking route by which Rip11 translocates to the plasma membrane in response to insulin we used total-internal reflection fluorescence (TIRF) microscopy. This technique, as deployed in our experiments, allows us to monitor the behaviour of any intracellular vesicles found within 90 nm of the plasma membrane and also any ensuing fusion events occurring between these two membranous compartments.

**Fig. 2:**
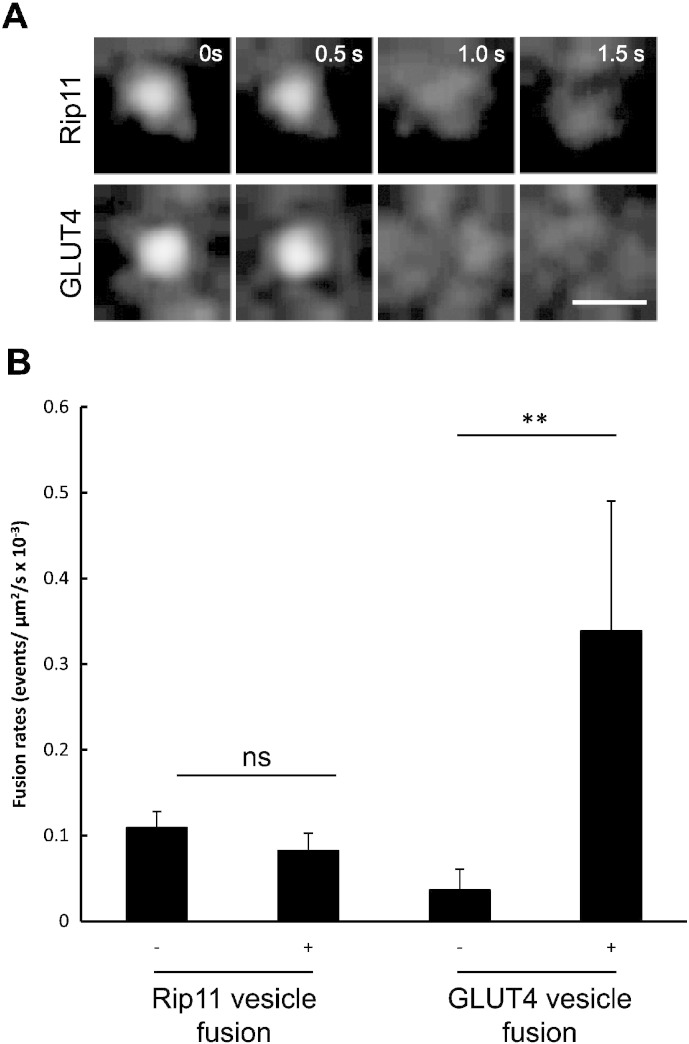
Insulin does not alter the rate of Rip11 vesicle fusion with the plasma membrane. 3T3–L1 adipocytes electroporated with plasmids encoding GFP–Rip11 or HA–GLUT4–GFP were serum starved and stimulated with or without insulin (87 nM) as indicated. (A) Cells were imaged live by TIRF microscopy at 2 frames/s using a penetration depth of 90 nm. Scale bar represents 1 μm. (B) The rate of vesicle fusion was calculated per μm^2^ membrane area per second. The graph represents mean ± SEM (N = 3, n = 8–343 fusion events, **p < 0.01, t-test, or not significant, ns).

In these experiments we found numerous highly dynamic GFP–Rip11 decorated vesicles in the region underlying the plasma membrane and thus within the TIRF zone. A sub-fraction of these vesicles underwent transient attachment to the plasma membrane prior to a rapid diffusion of the fluorescence away from the central point of vesicle attachment and into the membrane leaflet. The characteristics of these fusion events (Fig. 2A, top panels) were very similar to those of GFP-tagged GLUT4 vesicles that we, and others [29], [30], have demonstrated to undergo direct fusion with the plasma membrane leaflet (see Fig. 2A, lower panels).

In the basal state the fusion rate was calculated to be 1.09 ± 0.18 × 10^− 4^ events/μm^2^/s. This rate is similar to fusion rates observed for other recycling endosomal proteins, for example the transferrin receptor which exhibits 1.7 × 10^− 4^ fusion events/μm^2^/s in non-stimulated 3T3–L1 adipocytes [31], but almost three times higher than the basal fusion rate obtained for GLUT4 vesicles in these cells (0.40 ± 0.20 × 10^− 4^ events/μm^2^/s). The rate of GFP–Rip11 fusion was unchanged upon insulin stimulation, which contrasts with the more substantial increase in the rate of fusion of GLUT4 vesicles in response to insulin (Fig. 2B).

**Fig. 3:**
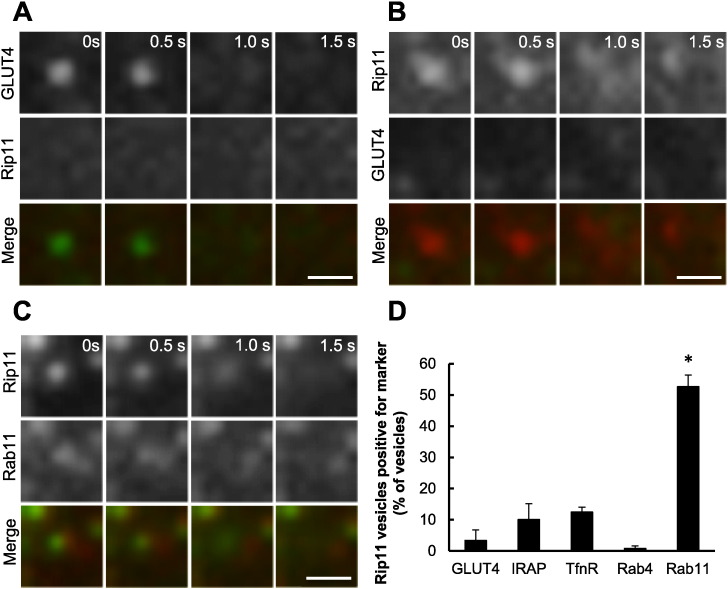
Rip11 vesicles fusing with the plasma membrane are positive for Rab11, but devoid of GLUT4 and IRAP. 3T3–L1 adipocytes electroporated with plasmids encoding mCherry–Rip11 and HA–GLUT4–GFP) (panels A and B), or GFP-Rip11 and mCherry.Rab11 (panel C) were stimulated with 87 nM insulin and examined by TIRF microscopy. Scale bar represents 1 μm. In panel (D) Rip11 fusion events were identified manually from images and examined for the presence of fluorescently-tagged GLUT4, IRAP, TfnR, Rab4 or Rab11. The graph represents mean ± SEM from three separate experiments (N = 3), in which 67–132 vesicles (‘n’) were examined from 4 to 5 cells per experiment. *p < 0.05 versus GLUT4, IRAP, transferrin receptor and Rab4 vesicle fusion rates.

The data suggest that insulin does not stimulate an increase in the amount of GFP–Rip11 at the plasma membrane via its ability to co-traffic with (or ‘piggy back’ on) insulin-regulated GLUT4 vesicles. This was confirmed using two-colour TIRF in which we imaged GFP–GLUT4 and a mCherry-tagged Rip11, which were co-expressed in the same 3T3–L1 adipocyte. In these experiments the majority of GLUT4 vesicles that we observed fusing with the plasma membrane were devoid of Rip11 (Fig. 3A). Similarly, the majority of fusing Rip11 vesicles were negative for GLUT4 (Fig. 3B and quantification in Fig. 3D), as well as IRAP, the transferrin receptor and Rab4 (Fig. 3D). By contrast, 52.6 ± 3.7% of the Rip11 fusing vesicles were positive for the known Rip11-binding partner, Rab11 (Fig. 3C and D).

The data suggest that Rip11 and GLUT4 traffic to the plasma membrane via distinct pathways, and that the effect of insulin on GFP–Rip11 translocation does not involve an increase in the rate of vesicle exocytosis or fusion with the plasma membrane.

### 3.3 Rip11 translocation does not require an interaction between its C2 domain and phosphoinositide lipids

Rip11 is found associated with intracellular vesicles, as well as in the cytosolic fraction of cells [16]. Rip11 also possesses a C2 domain that has been previously shown to bind to phosphoinositides, including PI(3,4,5)P_3_, and phosphatidic acid [14]. Furthermore, this domain has been previously shown to be important in phorbol ester-stimulated translocation of Rip11. The levels of PI(3,4,5)P_3_ are rapidly and substantially increased by insulin in the plasma membrane of 3T3–L1 adipocytes [32]. We asked, therefore, whether the interaction of Rip11 with PI(3,4,5)P_3_ was required for Rip11 translocation, perhaps via binding of a soluble pool of the protein to plasma membrane derived PI(3,4,5)P_3_ using a mechanism similar to that deployed by Akt, which possesses a PI(3,4,5)P_3_-binding Pleckstrin Homology (PH) domain [32].

**Fig. 4:**
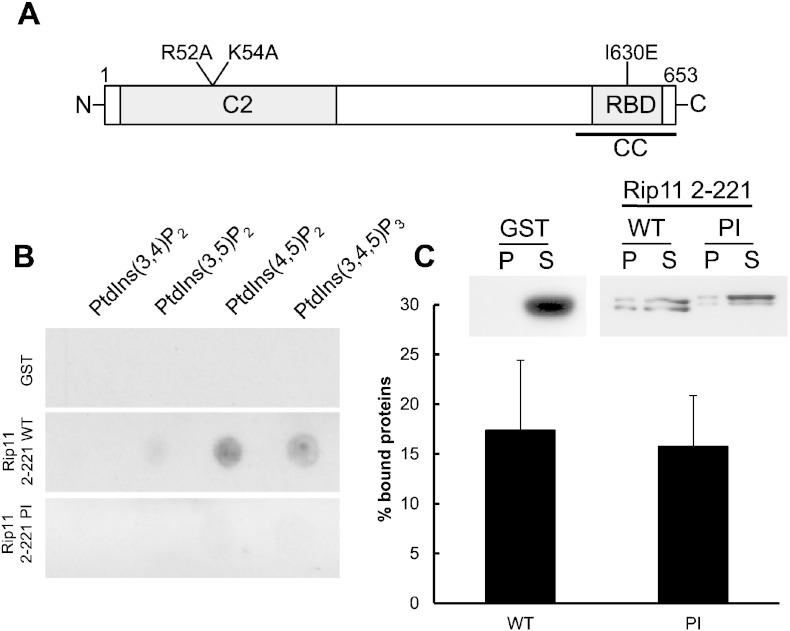
Identification of a surface phosphoinositide binding patch on Rip11 (A) Schematic diagram of Rip11 highlighting residues R52 and K54 which were mutated to alanines to generate a C2 domain mutant (the ‘PI’ mutant) which was incapable of binding to phosphoinositide lipids. (B) Recombinant GST–Rip11 C2 domain (amino acids 2–221; ‘WT’ or ‘PI’ mutant) or GST alone were incubated with PIP Strips™. Bound proteins were detected by blotting the membrane with an HRP-coupled anti-GST antibody. (C) The indicated purified recombinant proteins were incubated with phosphatidylcholine/phosphatidyl-ethanolamine (80:20) liposomes for 30 min at room temperature. Bound recombinant proteins were solubilised in sample buffer and resolved by SDS-PAGE before detection using an HRP-coupled anti-GST antibody. The graph represents mean ± SEM (N = 3).

We first sought to produce a mutant Rip11 that could not bind PI(3,4,5)P_3_. To do this we searched for basic residues in the Rip11 C2 domain that might be involved in phosphoinositide binding, by generating a homology model based on the published phosphoinositide-binding PKCα C2 domain structure [33]. Our model (VJJ & JMT, unpublished data) suggested that the Rip11 C2 domain residues most likely to be involved in PI(3,4,5)P_3_-binding were the surface-associated arginine-52 and lysine-54 (numbering according to NCBI NP_056285.1; Fig. 4A).

Recombinant wild-type Rip11 C2 domain (‘WT’; amino acids 2–221) and an equivalent C2 domain R52A/K54A mutant (‘PI’) were cloned and fused to GST for expression in, and purification from, *E. coli*. The ability of the purified recombinant proteins to bind phosphoinositide lipids was studied using a protein–lipid overlay assay as previously described [14]. As shown in Fig. 4B, the wild-type Rip11 C2 domain bound to PtdIns(4,5)P_2_ and PtdIns(3,4,5)P_3_, and to a lesser extent to PtdIns(3,5)P_2_. No binding was detected to PtdIns(3,4)P_2_. Strikingly, the R52A/K54A mutations (Rip11 ‘PI’ mutant) almost completely inhibited phosphoinositide-binding (Fig. 4B).

As the Rip11 C2 domain has also been shown to bind to the neutral phospholipids phosphatidylcholine (PC) and phosphatidylethanolamine (PE) [16], we wanted next to investigate the effect of the mutations on the binding to these lipids. In order to do so, we used a liposome binding assay, in which PC:PE liposomes (80:20) were incubated with purified recombinant proteins (Rip11 C2 domain, WT or the PI mutant). After sedimentation by centrifugation, bound proteins were analysed by Western blotting. As shown in Fig. 4C, binding to neutral liposomes was unaffected by the mutations. Taken together the results strongly suggest that basic residues R52 and K54 form a surface patch that is selectively involved in phosphoinositide-binding by the Rip11 C2 domain.

**Fig. 5:**
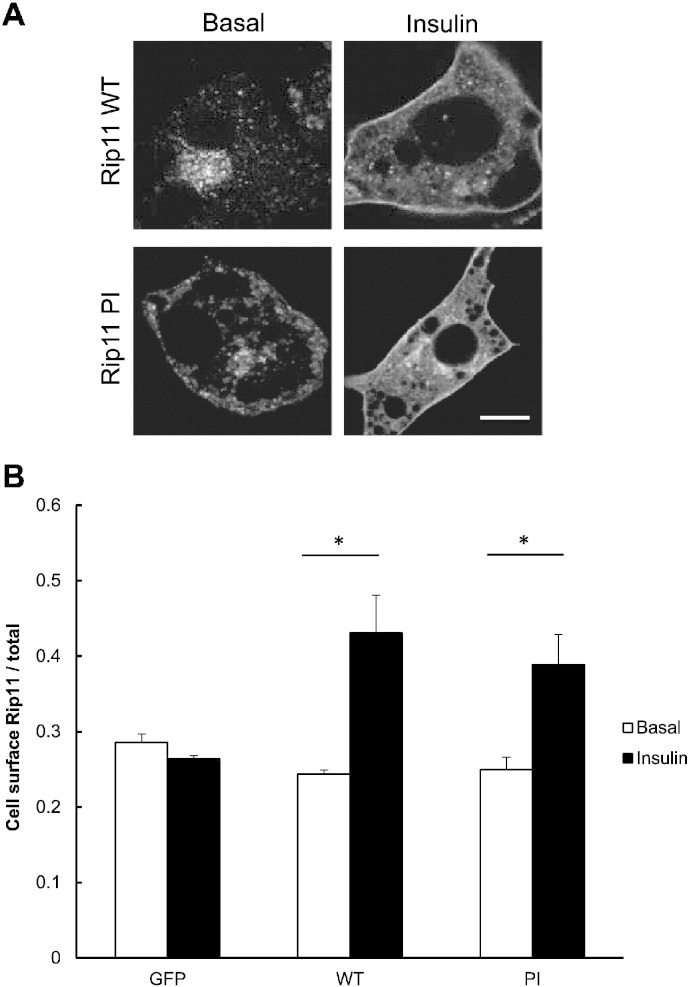
Phosphoinositide binding is not required for Rip11 translocation to the cell surface in response to insulin stimulation. 3T3–L1 adipocytes stably expressing GFP–Rip11 (WT or the PI mutant) were serum starved and stimulated with or without insulin before fixation. (A) Shows images of the cells treated in the absence or presence of insulin, as indicated, obtained by confocal microscopy. Scale bar represents 10 μm. (B) The graph represents mean ± SEM of the amount of Rip11 at the cell surface normalised to total Rip11 expression. (N = 3, n = 30 cells). *p < 0.05, t-test.

We next analysed the effect of the mutations on the translocation of GFP–Rip11 to the plasma membrane in response to insulin in 3T3–L1 adipocytes. In the basal state, wild-type GFP–Rip11 and the GFP–Rip11–PI mutant localised to the perinuclear region of the cell and to peripheral vesicles, but were largely excluded from the plasma membrane (Fig. 5A) suggesting that phosphoinositide-binding is not necessary for Rip11 targeting to intracellular membranes. After insulin stimulation, we found that the GFP–Rip11–PI mutant accumulated at the plasma membrane in a manner indistinguishable from the wild-type protein (Fig. 5A and B). The data suggest that phosphoinositide-binding is not required for Rip11 translocation to the plasma membrane.

### 3.4 Inhibition of dynamin function mimics insulin-stimulated Rip11 translocation

The studies above suggest that insulin does not stimulate Rip11 translocation to the plasma membrane via the binding of Rip11 to plasma membrane derived phosphoinositide lipids such as PI(3,4,5)P_3_ or through an increase in the rate of Rip11 vesicle exocytosis. Given that insulin has been reported to inhibit GLUT4 endocytosis in adipocytes [34], [35] we next asked whether an inhibition of Rip11 endocytosis by insulin might be the underlying cause of the apparent translocation we observe.

**Fig. 6:**
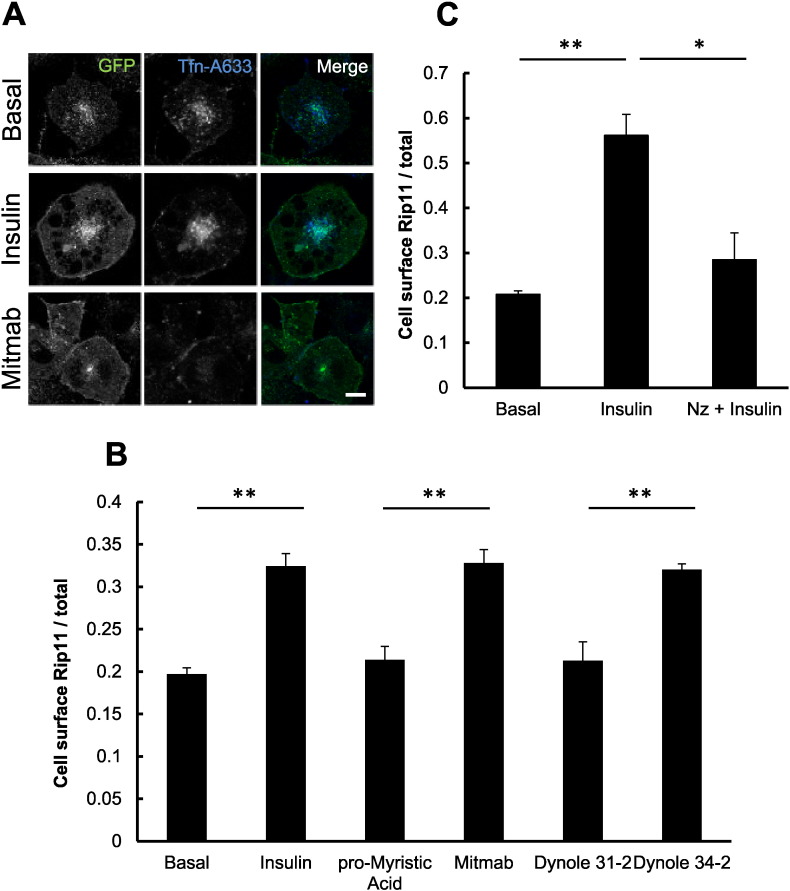
Inhibition of dynamin-dependent endocytosis induces Rip11 accumulation at the plasma membrane. 3T3–L1 adipocytes expressing GFP–Rip11 were serum starved and stimulated with the following compounds: 40 μM Mitmab, 40 μM pro-Myristic acid, 10 μM Dynole 34–2, 10 μM Dynole 31–2 or 10 μM nocodazole, in the presence or absence of 87 nM insulin, as indicated. (A) Cells were labelled with fluorescently-tagged transferrin (25 μg/ml) for 30 min in the presence and absence of insulin or Mitmab, fixed and imaged by confocal microscopy. Scale bar represents 10 μm. (B) Quantification of amount of Rip11 at the cell surface in the presence or absence of dynamin inhibitors as indicated. (C) Cells were incubated with or without nocodazole (Nz) for 30 min followed by stimulation with or without insulin in the continuous presence of nocodazole before fixation and immunostaining. The histogram represent mean ± SEM (N = 3, n = 20 cells) with *p < 0.05, **p < 0.01, (t-test).

To explore this, we used the dynamin inhibitor Mitmab. Dynamin plays a key role in both clathrin- [36] and caveolin-dependent [37] endocytosis of cargo, including, for example, the transferrin receptor. As shown in Fig. 6A, and as quantified in Fig. 6B, Mitmab mimicked the effect of insulin on GFP–Rip11 translocation to the plasma membrane, while at the same time it almost completely blocked the uptake of fluorescent transferrin (Fig. 6A), as expected. Importantly, the inactive analogue of Mitmab (pro-myristic acid) was without effect on GFP–Rip11 translocation (Fig. 6B). To further confirm the specificity of the effect, we used Dynole-34-2, a structurally dissimilar inhibitor of dynamin-dependent endocytosis and its inactive analogue Dynole 31–2 [38]. Again, Dynole 34–2 almost completely mimicked the effect of insulin, while its inactive analogue was without effect (Fig. 6B).

Taken together the data suggest that insulin promotes GFP–Rip11 accumulation in the plasma membrane through an inhibition of endocytosis, rather than by stimulating GFP–Rip11 vesicle exocytosis. Interestingly, we had noted that nocodazole, which inhibits vesicle exocytosis by promoting the depolymerisation of microtubules, blocks the effect of insulin on Rip11 accumulation in the plasma membrane (Fig. 6C). This is consistent with the requirement for continuous GFP–Rip11 recycling for insulin to have any effect.

### 3.5 Insulin inhibits Rip11 endocytosis in a PI 3-kinase-dependent and Akt-independent manner

**Fig. 7:**
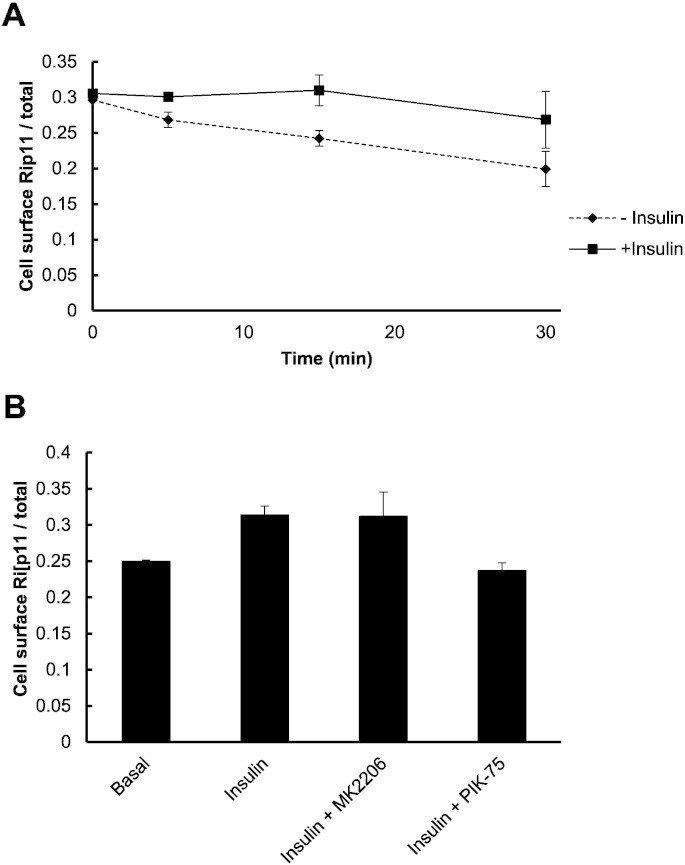
Insulin inhibits internalisation of Rip11 from the cell surface in a PI 3-kinase dependent manner. 3T3–L1 adipocytes stably expressing GFP–Rip11 were serum starved and incubated with 40 μM Mitmab for 30 min. In (A) the cells were washed in ice-cold PBS to remove Mitmab and then incubated at 37 °C for the indicated times in the continuous presence (squares) or absence (diamonds) of insulin. The amount of cell surface Rip11 was quantified from the images obtained by confocal microscopy. In (B) the cells were washed in ice-cold PBS and then incubated at 37 °C for 30 min in the absence (basal) or continuous presence of insulin, MK2206 or PIK-75, as indicated. The histogram represents mean ± SEM (N = 2, n = 25 cells).

To confirm that insulin directly affects Rip11 endocytosis, we performed a ‘Mitmab washout’ experiment, taking advantage of the fact that Mitmab is a reversible dynamin inhibitor [36]. Cells were first incubated with Mitmab to promote the accumulation of GFP–Rip11 in the plasma membrane. The cells were then washed to remove the Mitmab (t = 0 in Fig. 7A), before continuing the incubation in the absence or presence of insulin. Cells were fixed at 5, 15 and 30 min and the rate at which GFP–Rip11 re-internalised was measured. We reasoned that if insulin inhibited GFP–Rip11 endocytosis, then the rate at which GFP–Rip11 is removed from the plasma membrane should be slowed. As shown in Fig. 7A, we did indeed observe that the level of plasma membrane associated GFP–Rip11 fell in the absence of insulin and that this fall was slowed in its presence.

As the insulin-stimulated accumulation of GFP–Rip11 in the plasma membrane was shown to be PI 3-kinase dependent but independent of Akt activity (Fig. 1B), we next wanted to confirm that this was also the case for the ability of insulin to inhibit GFP–Rip11 endocytosis. Cells were incubated in Mitmab to promote an increased level of cell surface GFP–Rip11 and washed to then remove the Mitmab. The cells were then further incubated in the presence of insulin, but with or without the addition of MK2206 or PIK-75. As shown in Fig. 7B, the ability of insulin to slow GFP–Rip11 endocytosis was completely prevented by the PI 3-kinase inhibitor, PIK-75. By contrast, the Akt inhibitor MK2206 was without effect. These collective observations are consistent with the data shown in Fig. 1B.

## 4 Discussion

It is well established that Rip11 plays an important role in regulating the trafficking of cargo between early endosomes and recycling endosomes [15], and between the endosomal recycling compartment and the plasma membrane [14], [16]. Rip11 has also been demonstrated to translocate from an intracellular compartment(s) to the plasma membrane in response to insulin and phorbol esters [14], [21], and in this study we have explored the mechanism of this effect of insulin in greater detail using the 3T3–L1 adipocyte model system.

We initially reasoned that the stimulus-induced increase in abundance of Rip11 at the plasma membrane could occur through enhanced delivery of Rip11 on exocytic vesicle carriers, in particular those also containing the insulin-regulated GLUT4 glucose transporter. The rate of Rip11 vesicle fusion with the plasma membrane was measured using TIRF microscopy, but this was unchanged in response to insulin (Fig. 2B), unlike the increased fusion of the insulin-regulated cargo, GLUT4. This strongly suggests that the movement of Rip11 towards the cell surface on membrane carriers is unaffected by insulin, and that Rip11 does not translocate to the plasma membrane by ‘piggy-backing’ on GLUT4 vesicles.

We further considered a role for the binding of a soluble cytosolic pool of Rip11 with the plasma membrane via its phospholipid binding C2 domain. Using molecular modelling, we identified a putative phosphoinositide-binding patch on the surface of the Rip11 C2 domain and which comprised the basic amino acids arginine-52 and lysine-54. A Rip11–R52A/K54A mutant (the ‘PI’ mutant) was unable to bind phosphoinositides lipids (Fig. 4B) but could translocate to the plasma membrane in response to insulin in a manner indistinguishable from the wild-type protein (Fig. 5). This makes it unlikely that insulin brings about the translocation of Rip11 via a fluid phase movement of soluble GFP–Rip11 to the plasma membrane where it might bind, for example, PI(3,4,5)P_3_ produced in response to insulin (as occurs with insulin-stimulated Akt translocation). This contrasts with phorbol ester-stimulated Rip11 translocation which occurs in a manner that can be prevented by deletion of the C2 domain [14].

We then explored whether dynamin-mediated endocytosis played a role in Rip11 internalisation through the use of two structurally dissimilar cell-permeant small molecule dynamin inhibitors, Mitmab and Dynole 34-2. Mitmab and Dynole 34-2 both mimicked the effect of insulin on GFP–Rip11 translocation (Fig. 6A and B), while their inactive structurally related analogues were without effect. A subsequent ‘Mitmab-washout’ experiment confirmed that insulin inhibited the internalisation of GFP–Rip11 (Fig. 7). Taken together with the fact that nocodazole inhibited the insulin dependent appearance of GFP–Rip11 at the plasma membrane (Fig. 6C), we propose a model in which, in the basal state, the relatively high rate of recycling of GFP–Rip11 maintains a continuous flow of Rip11 arriving at the plasma membrane from recycling Rab11-positive endosomes, which is matched by a continuous re-internalisation of GFP–Rip11 from the plasma membrane in a manner that requires dynamin. This provides for a steady state situation where the majority of the GFP–Rip11 is located in juxtanuclear recycling endosomes and only a relatively small proportion (approx. 20% according to Fig. 1B) is found at or near the plasma membrane. We suggest, therefore, that insulin signalling causes the inhibition of GFP–Rip11 internalisation through suppression of a dynamin-dependent process, which results in a net accumulation of Rip11 at the plasma membrane.

The precise mechanism by which Rip11 internalises from the plasma membrane requires further investigation. It could occur via its direct recruitment onto endocytic carriers, which are generated through the action of dynamin. Alternatively, Rip11 could be indirectly recycled between the cell interior and plasma membrane by virtue of its interaction with a binding partner that is itself the recycled cargo. We believe it unlikely that this binding partner is either of Rab11 or Rab14, which are the only known Rip11 interacting Rab proteins because: (i) GFP–Rab11 and GFP–Rab14 do not translocate to the plasma membrane of 3T3–L1 adipocytes in response to insulin (F.B., S.E.R. & J.M.T., unpublished observations) and (ii) a Rip11[I630E] mutant, which cannot bind either Rab11 or Rab14 [39], still exhibited a pronounced translocation to the plasma membrane in response to insulin [21].

Interestingly, Rip11 internalisation shares several characteristics of the insulin-regulated cargo, GLUT4. The translocation of GLUT4 to the cell surface in response to insulin is predominantly the result of an increase in the trafficking of the transporter from a specialised GLUT4 storage compartment, however the hormone has also been shown to have an inhibitory effect on GLUT4 endocytosis, particularly in adipocytes [34], [40], [41], [42], [43], [44]. The inhibition of GLUT4 internalisation by insulin is PI 3-kinase dependent but Akt-independent [42], [45]. Furthermore, GLUT4 internalisation occurs at least in part via a dynamin-dependent pathway in adipocytes and muscle cells [46], [47], [48]. This suggests that Rip11 internalisation could indeed occur by co-trafficking with GLUT4 on endocytic carriers. We were unable to observe GLUT4 endocytosis using TIRF microscopy, so this possibility requires further future investigation.

It is well established that Rip11 plays a central role in directing the trafficking of cargo between endosomes and recycling endosomes [15] and between endosomal compartments and the plasma membrane [14], [16], so the reason why Rip11 accumulates at the plasma membrane in response to stimuli such as insulin and phorbol esters is poorly understood. In polarised MDCK cells Rip11 has been reported to be important for trafficking IgA from supranuclear apical recycling endosomes to the plasma membrane through a mechanism likely to involve Rab11 [16]. In adipocytes, we have reported that Rip11 plays a role as a scaffolding protein in the fusion of intracellular GLUT4 vesicles with the plasma membrane [21], rather than in their translocation from intracellular compartments. An insulin-dependent accumulation of Rip11 in the plasma membrane as a result of an inhibition of dynamin-dependent Rip11 endocytosis, could bring Rip11 into juxtaposition with fusing GLUT4 vesicles whereby it then controls the fusion process.

To conclude, our current study demonstrates that insulin promotes Rip11 translocation to the plasma membrane via a dynamin- and PI 3-kinase-dependent, but Akt-independent, route. Given the similarities between the mechanism of insulin-regulated GLUT4 endocytosis, about which relatively little is known, and that of Rip11, further studies of these related phenomena are clearly warranted.

## Conflict of interest

The authors declare no conflict of interest.

## References

[1] Hutagalung A.H., Novick P.J. (2011). Physiol. Rev..

[2] Stenmark H. (2009). Nat. Rev. Mol. Cell Biol..

[3] Grosshans B.L., Ortiz D., Novick P. (2006). Proc. Natl. Acad. Sci. U. S. A..

[4] Bucci C., Parton R.G., Mather I.H., Stunnenberg H., Simons K., Hoflack B., Zerial M. (1992). Cell.

[5] Chavrier P., Parton R.G., Hauri H.P., Simons K., Zerial M. (1990). Cell.

[6] Gorvel J.P., Chavrier P., Zerial M., Gruenberg J. (1991). Cell.

[7] van der Sluijs P., Hull M., Webster P., Male P., Goud B., Mellman I. (1992). Cell.

[8] Ullrich O., Reinsch S., Urbe S., Zerial M., Parton R.G. (1996). J. Cell Biol..

[9] Hales C.M., Griner R., Hobdy-Henderson K.C., Dorn M.C., Hardy D., Kumar R., Navarre J., Chan E.K., Lapierre L.A., Goldenring J.R. (2001). J. Biol. Chem..

[10] Lindsay A.J., McCaffrey M.W. (2002). J. Biol. Chem..

[11] Prekeris R. (2003). ScientificWorldJournal.

[12] Prekeris R., Davies J.M., Scheller R.H. (2001). J. Biol. Chem..

[13] Wallace D.M., Lindsay A.J., Hendrick A.G., McCaffrey M.W. (2002). Biochem. Biophys. Res. Commun..

[14] Lindsay A.J., McCaffrey M.W. (2004). J. Cell Sci..

[15] Schonteich E., Wilson G.M., Burden J., Hopkins C.R., Anderson K., Goldenring J.R., Prekeris R. (2008). J. Cell Sci..

[16] Prekeris R., Klumperman J., Scheller R.H. (2000). Mol. Cell.

[17] Willenborg C., Prekeris R. (2011). Biosci. Rep..

[18] Schwenk R.W., Luiken J.J., Eckel J. (2007). Biochem. Biophys. Res. Commun..

[19] Sugawara K., Shibasaki T., Mizoguchi A., Saito T., Seino S. (2009). Genes Cells.

[20] Oehlke O., Martin H.W., Osterberg N., Roussa E. (2010). J. Cell. Physiol..

[21] Welsh G.I., Leney S.E., Lloyd-Lewis B., Wherlock M., Lindsay A.J., McCaffrey M.W., Tavare J.M. (2007). J. Cell Sci..

[22] Arcaro A., Wymann M.P. (1993). Biochem. J..

[23] Wymann M.P., Bulgarelli-Leva G., Zvelebil M.J., Pirola L., Vanhaesebroeck B., Waterfield M.D., Panayotou G. (1996). Mol. Cell. Biol..

[24] Hirai H., Sootome H., Nakatsuru Y., Miyama K., Taguchi S., Tsujioka K., Ueno Y., Hatch H., Majumder P.K., Pan B.S., Kotani H. (2010). Mol. Cancer Ther..

[25] Danson C.M., Pocha S.M., Bloomberg G.B., Cory G.O. (2007). J. Cell Sci..

[26] Zufferey R., Nagy D., Mandel R.J., Naldini L., Trono D. (1997). Nat. Biotechnol..

[27] Boal F., Le Pevelen S., Cziepluch C., Scotti P., Lang J. (2007). Biochim. Biophys. Acta.

[28] Knight Z.A., Gonzalez B., Feldman M.E., Zunder E.R., Goldenberg D.D., Williams O., Loewith R., Stokoe D., Balla A., Toth B., Balla T., Weiss W.A., Williams R.L., Shokat K.M. (2006). Cell.

[29] Huang S., Lifshitz L.M., Jones C., Bellve K.D., Standley C., Fonseca S., Corvera S., Fogarty K.E., Czech M.P. (2007). Mol. Cell. Biol..

[30] Lizunov V.A., Matsumoto H., Zimmerberg J., Cushman S.W., Frolov V.A. (2005). J. Cell Biol..

[31] Xu Y., Rubin B.R., Orme C.M., Karpikov A., Yu C., Bogan J.S., Toomre D.K. (2011). J. Cell Biol..

[32] Oatey P.B., Venkateswarlu K., Williams A.G., Fletcher L.M., Foulstone E.J., Cullen P.J., Tavare J.M. (1999). Biochem. J..

[33] Guerrero-Valero M., Ferrer-Orta C., Querol-Audi J., Marin-Vicente C., Fita I., Gomez-Fernandez J.C., Verdaguer N., Corbalan-Garcia S. (2009). Proc. Natl. Acad. Sci. U. S. A..

[34] Karylowski O., Zeigerer A., Cohen A., McGraw T.E. (2004). Mol. Biol. Cell.

[35] Yang J., Holman G.D. (1993). J. Biol. Chem..

[36] Quan A., McGeachie A.B., Keating D.J., van Dam E.M., Rusak J., Chau N., Malladi C.S., Chen C., McCluskey A., Cousin M.A., Robinson P.J. (2007). Mol. Pharmacol..

[37] Hinshaw J.E. (2000). Annu. Rev. Cell Dev. Biol..

[38] Hill T.A., Gordon C.P., McGeachie A.B., Venn-Brown B., Odell L.R., Chau N., Quan A., Mariana A., Sakoff J.A., Chircop M., Robinson P.J., McCluskey A. (2009). J. Med. Chem..

[39] Kelly E.E., Horgan C.P., Adams C., Patzer T.M., Ni Shuilleabhain D.M., Norman J.C., McCaffrey M.W. (2010). Biol. Cell..

[40] Blot V., McGraw T.E. (2006). EMBO J..

[41] Czech M.P., Buxton J.M. (1993). J. Biol. Chem..

[42] Fazakerley D.J., Holman G.D., Marley A., James D.E., Stockli J., Coster A.C. (2010). J. Biol. Chem..

[43] Jhun B.H., Rampal A.L., Liu H., Lachaal M., Jung C.Y. (1992). J. Biol. Chem..

[44] Zeigerer A., McBrayer M.K., McGraw T.E. (2004). Mol. Biol. Cell.

[45] Gonzalez E., McGraw T.E. (2006). Mol. Biol. Cell.

[46] Volchuk A., Narine S., Foster L.J., Grabs D., De Camilli P., Klip A. (1998). J. Biol. Chem..

[47] Kao A.W., Ceresa B.P., Santeler S.R., Pessin J.E. (1998). J. Biol. Chem..

[48] Al-Hasani H., Hinck C.S., Cushman S.W. (1998). J. Biol. Chem..

